# Iminosugars With Endoplasmic Reticulum α-Glucosidase Inhibitor Activity Inhibit ZIKV Replication and Reverse Cytopathogenicity *in vitro*

**DOI:** 10.3389/fmicb.2020.00531

**Published:** 2020-04-07

**Authors:** Gitanjali Bhushan, Levina Lim, Ian Bird, Shubhada K. Chothe, Ruth H. Nissly, Suresh V. Kuchipudi

**Affiliations:** Penn State Animal Diagnostic Laboratory, Department of Veterinary and Biomedical Sciences, Pennsylvania State University, University Park, PA, United States

**Keywords:** Zika virus, antiviral, iminosugar, ER-AGI, castanospermine, celgosivir, DNJ

## Abstract

Zika virus (ZIKV), a vector-borne virus of the family *Flaviviridae*, continues to spread and remains a significant global public health threat. Currently, there are no approved vaccines or antivirals against ZIKV. We investigated the anti-ZIKV ability of three iminosugars with endoplasmic reticulum α-glucosidase inhibitor (ER-AGI) activity, namely deoxynojirimycin (DNJ), castanospermine, and celgosivir. None of the three iminosugars showed any significant cytotoxicity in Vero or human microglia CHME3 cells when applied for 72 h at concentrations up to 100 μM. Iminosugar treatment of Vero or CHME3 cells prior to ZIKV infection resulted in significant inhibition of ZIKV replication over 48 h. Reduction in ZIKV replication in iminosugar-treated cells was not associated with any significant change in the expression levels of key antiviral genes. Following infection with three different strains of ZIKV, iminosugar-treated Vero or CHME3 cells showed no cell death, whereas vehicle-treated control cells exhibited 50–60% cell death at 72 h post-infection (hpi). While there was no significant difference in apoptosis between iminosugar-treated and control cells, iminosugar-treated cells exhibited a substantial reduction of necrosis at 72 hpi following ZIKV infection. In summary, iminosugars with ER-AGI activity inhibit ZIKV replication and significantly reduce necrosis without altering the antiviral gene expression and apoptosis of infected human cells. The results of this study strongly suggest that iminosugars are promising anti-ZIKV antiviral agents and such warrant further *in vivo* studies.

## Introduction

Zika virus is an icosahedral, enveloped RNA virus of the *Flaviviridae* family and flavivirus genus. *Aedes aegypti* and *Aedes albopictus* mosquito species are the two main insect vectors that transmit ZIKV (Vorou, 2016). Human to human transmission through bodily fluids and the maternal-fetal route has also been established (WHO, 2016). Zika virus (ZIKV) has recently garnered global attention following its re-emergence in the past decade throughout the Pacific Islands and the Americas. In February 2016, the World Health Organization (WHO) declared a public health emergency due to the increased association of ZIKV infections with fetal abnormalities, autoimmune disease, and neurological disorders. ZIKV has been relatively uninvestigated since its initial discovery in Uganda in 1947 (Dick et al., 1952) and only came to public attention in 2013 when several humans were infected with ZIKV in French Polynesia (Cao-Lormeau et al., 2014; Oehler et al., 2014). Until this point, symptoms of ZIKV infection were mild, including fever, rash, and malaise. Since its emergence in the Pacific islands and the Americas, ZIKV has been associated with increased infection rates, neurological pathologies such as Guillain-Barré syndrome, meningoencephalitis, and myelitis in adults, and microcephaly in infants (Musso and Gubler, 2016).

By phylogenetic analysis, ZIKV isolates cluster into two lineages, namely African and Asian (Haddow et al., 2012). Epidemic ZIKV strains from French Polynesia and the Americas cluster are included in the Asian lineage of ZIKV (Lanciotti et al., 2016). African strains have been shown to induce a more cytopathic effect *in vitro* in comparison to Asian strains (Anfasa et al., 2017; Bhatnagar et al., 2017; Yuan et al., 2017; Sheridan et al., 2018). It is speculated that Asian strains induce less cytopathic effect and maintain cell viability to allow a longer period of viral persistence and replication (Sheridan et al., 2018).

One of the most alarming outcomes associated with ZIKV infection during pregnancy is microcephaly (Petersen et al., 2016). While research is still ongoing to understand the relationship, Asian lineage ZIKVs have been most associated with microcephaly (Anfasa et al., 2017; Bhatnagar et al., 2017; Yuan et al., 2017; Majumder et al., 2018; Sheridan et al., 2018; Jaeger et al., 2019; Udenze et al., 2019). Microcephaly is a condition in which fetuses are born with small heads due to abnormal brain development. ZIKV RNA has been found in amniotic fluid as well as the brain of fetuses and infants with microcephaly (Oliveira Melo et al., 2016; Mlakar et al., 2016). In addition to placental cells such as Hofbauer macrophages and trophoblasts, fetal brain cells are targets of ZIKV infection (Kendra et al., 2016). *In vitro* studies have shown that neural progenitor cells, astrocytes, microglia, and oligodendrocyte precursor cells are vulnerable to ZIKV infection (Tang et al., 2016; Retallack et al., 2016). An ability to limit ZIKV replication in mothers and/or reduce ZIKV infection of fetal brain cells may prevent microcephaly.

Current strategies for the prevention and control of ZIKV involves vector control and symptomatic therapy. Despite the considerable need for novel antiviral therapies, currently, there are no FDA-approved drugs to prevent and treat ZIKV infection. A primary focus of current ZIKV antiviral research is directed at targeting virus entry and the virus replication pathways. ZIKV entry is mediated by a set of proposed receptors such as T-cell immunoglobulin and mucin domain (TIM) and TYRO-3, AXL, and MERTK (TAM) families (Richard et al., 2017). However, it is unclear whether there are additional and/or alternative receptors that facilitate ZIKV entry. A significant downside of virus-directed antiviral agents in the development of resistance, especially in the case of RNA viruses that have a high mutation rate. Hence, a combination of virus-directed and host-directed antivirals could be a more practical approach for ZIKV antiviral therapy.

A promising avenue for effective anti-flaviviral therapeutics is a class of host-directed antivirals, namely iminosugars, with ER α-glucosidase inhibitor (ER-AGI) activity that are known to inhibit a range of enveloped RNA and DNA viruses *in vitro* by interrupting proper folding of viral proteins (Mehta et al., 1998; Chang et al., 2013a, b; Perry et al., 2013; Alonzi et al., 2017; Ma et al., 2018). Iminosugars are sugar mimetics in which cyclic oxygen is replaced with nitrogen. They mimic endogenous sugars and compete with endogenous substrates for binding to ER α-glucosidases. ER α-glucosidases I and II are responsible for trimming terminal glucose moieties on N-linked glycans attached to nascent glycoproteins. α-glucosidase I removes the outermost α-1,2-linked glucose residue while α-glucosidase II removes the inner two α-1,3-linked glucose residues. These steps are essential for subsequent calnexin/calreticulin chaperone interaction (Whitby et al., 2005). Incompletely folded proteins are re-glycosylated by UDP-glucose: glycoprotein glucosyltransferase (UGGT) and undergo the process again until they are properly folded. While properly folded glycoproteins move to the Golgi apparatus for maturation, improperly folded glycoproteins accumulate in the ER and will ultimately undergo ER-associated degradation (ERAD) (Chang et al., 2013b). In studies, patients deficient in α-glucosidases I or II showed no clinical evidence of recurrent viral infections, and cells derived from these patients were unable to support infection by multiple viruses such as HIV, influenza A virus, adenovirus, poliovirus and vaccinia virus (Sadat et al., 2014; Alonzi et al., 2017).

Zika virus, like other flaviviruses, has three N-glycosylated proteins, precursor of the membrane (prM), envelope (E), and non-structural protein NS1, that are substrates for the ER α-glucosidase enzymes during ZIKV replication (Wu et al., 2002). ZIKV replication and viral RNA synthesis occurs on an extended network of modified endoplasmic reticulum (ER) membranes. The ZIKV capsid protein binds to viral RNA during the process of nucleocapsid assembly, forming the core of mature virus particles, which is aided by NS1 protein (Apte-Sengupta et al., 2014). The immature virus particles pass through the *trans-*Golgi-network to undergo maturation by conformational changes in the E protein and furin cleavage of the prM protein (Apte-Sengupta et al., 2014; Meertens et al., 2017).

Although iminosugars seem to be effective antivirals against many flaviviruses, only one recent report has examined the role of these compounds in ZIKV infection (Ma et al., 2018). In these studies, hepatocellular carcinoma cells with the knockout of α-glucosidases I or II supported a reduced level of ZIKV viral replication compared with wild-type cells (Ma et al., 2018). ER α-glucosidases I was more critical in reducing virus replication in comparison to ER α-glucosidase II (Ma et al., 2018). ZIKV viral replication was reduced in embryonic kidney cells when cells were treated with the iminosugar IVHR-19029 (Ma et al., 2018). To date, no investigations of the use of iminosugars against ZIKV in clinically relevant cell types, such as human brain cells, have been reported. There is no information about potential differential sensitivity to iminosugars between African or Asian lineage ZIKVs.

Here, we report the ability of iminosugars comprising a monocyclic amine deoxynojirimycin (DNJ) and bicyclic amines castanospermine and celgosivir that possess ER-AGI activity to inhibit replication of multiple strains of ZIKVs from different lineages *in vitro* in both Vero cells and human microglia CHME3 cells. Iminosugar treatment resulted in a significant reduction in ZIKV replication and cell necrosis. Notably, iminosugar treatment did not alter apoptosis and antiviral response of ZIKV infected cells.

## Materials and Methods

### Viruses, Cells, and Compounds

Two human ZIKV strains, namely PRVABCB59 (Human/2015/Puerto Rico), IBH30656 (Human/1968/Nigeria) and one mosquito ZIKV strain MEX 2-81 (Mosquito/2016/Mexico) obtained from BEI Resources were used in this study. All viruses were propagated in Vero cells, and virus titration was carried out by TCID_50_ in Vero and calculated using the method of Reed and Muench (Reed and Muench, 1938). African green monkey kidney Vero (CCL-81) cells were purchased from ATCC and cultured in medium containing Dulbecco’s modified Eagle’s medium (DMEM; Corning, NY, United States) supplemented with 10% heat-inactivated fetal bovine serum (FBS; Corning) and 1% antibiotic/antimycotic solution (Corning) at 37°C with 5% CO_2_. Human immortalized microglial cell lines (CHME3) cells (courtesy Dr. Pamela Hankey Giblin, Penn State University) were used in this study. CHME3 cell line was established through SV40-dependent immortalization of a human fetal brain-derived primary microglia culture. Human microglial cell line CHME3 was cultured in medium containing Dulbecco’s modified Eagle’s medium (DMEM; Corning) supplemented with 10% heat-inactivated fetal bovine serum (FBS; Corning), 1% antibiotic/antimycotic solution (Corning), and 0.1% non-essential amino acids (NEAA, GE Life Sciences, Marlborough, MA, United States) at 37°C with 5% CO_2_. ER α-glucosidase inhibitors castanospermine and 1-deoxynojirimycin hydrochloride (DNJ) were purchased from Sigma-Aldrich (St. Louis, MO, United States), and celgosivir was purchased from MedChem Express (Monmouth Junction, NJ, United States). All compounds were dissolved in sterile nuclease-free water.

### Cytotoxicity and Antiviral Screening Assay

A cell viability assay was used to evaluate both the cytotoxicity and antiviral abilities of the iminosugars with appropriate controls as previously described (Fletcher et al., 2000) using the CellTiter 96^®^ AQueous One Solution Cell Proliferation Assay (MTS, Promega, Madison, WI, United States) following the manufacturer’s instructions. At 60–70% confluence, Vero cells and CHME3 cells were pretreated with iminosugars for 2 h and then infected with ZIKV according to the plate layout shown in Supplementary Figure S1. Four biological replicates were used to assess cytotoxicity, and six replicates were used to assess the antiviral ability of each iminosugar with concentrations ranging from 0.01 to 1000 μM or vehicle (sterile nuclease-free water). Cells treated with iminosugars or vehicle control were infected with ZIKV strain PRVABC59, IBH30656 or MEX 2-81 at a multiplicity of infection (MOI) of 1 in infection medium consisting of DMEM supplemented with 5% FBS and 1% antibiotic/antimycotic solution. The CHME3 infection medium was additionally supplemented with 0.1% non-essential amino acids (NEAA). At 72 h post-infection (hpi), MTS solution was added to the wells, and after 2 h, the optical density was measured at 490 nm using a microplate reader (ELx800; BioTek, Winooski, VT, United States). Previous studies have shown that ER α-glucosidase inhibitor, Celgosivir prevents DENV secretion in primary human macrophages with an EC_50_ of 5 μM and has CC_50_ greater than 1000 μM in MBDK cells (Whitby et al., 2004; Sung et al., 2016).

### Virus Yield Reduction Assay

Zika virus yield reduction following treatment with iminosugars was assessed by measuring the NS1 gene by a previously described quantitative reverse transcription-polymerase chain reaction (qRT-PCR) and infectious virus titration in Vero cells (Reed and Muench, 1938; Goebel et al., 2016). Vero and CHME3 cells pre-treated 1 μM of castanospermine, celgosivir or DNJ for 2 h and then were infected with PRVABC59 either at MOI 0.5 or 5 in infection medium. After 2 h of incubation with the virus, medium from cells was removed, cells were washed with phosphate buffered saline, and fresh infection medium containing 1 μM of castanospermine, celgosivir or DNJ was added to the wells. At 48 and 72 hpi, culture supernatants were harvested and stored at −80°C until use for viral RNA extraction. Viral RNA was extracted from 50 μl of sample using the MagMAX-96 Viral RNA Isolation Kit (Applied Biosystems, Foster City, CA, United States) on a MagMAX Express-96 Deep Well Magnetic Particle Processor (Applied Biosystems). The amount of ZIKV genomic RNA was measured by qRT-PCR using SuperScript^®^ III Platinum One-Step qRT-PCR Kit on a 7500 Fast Real-Time PCR System (Applied Biosystems). The primers (F: 5′-ATATCGGACATGGCTTCGGA-3′, R: 5′-GTTCTTTTACAGACATATTGAGTGTC-3′) and probe sequence (5′- FAM-TGCCCAACA/ZEN/CAAGGTGAAGCC TACCT-BHQ) were purchased from Integrated DNA Technologies (IDT; Coralville, IA, United States), and the qRT-PCR cycling conditions included an initial cDNA synthesis for 30 min at 50°C, followed by 2 min at 95°C and 45 cycles of 2-step cycling at 95°C for 15 s, then 55°C for 30 s (Goebel et al., 2016). The ZIKV copy numbers were quantified using a standard curve generated with serial dilutions of positive control ZIKV RNA standards derived from ZIKV with known TCID_50_ value. Cell culture supernatants were titrated for the infectious virus in Vero cells, and titer was expressed as TCID_50_ calculated using the Reed-Muench method (Reed and Muench, 1938).

### Measurement of Apoptosis

Apoptotic cell death was evaluated by measuring the levels of active caspase 3 and 7 in cells using the Caspase-Glo^®^ 3/7 Assay (Promega) following the manufacturer’s instructions. CHME3 cells in 96-well white-walled cell culture plates (Greiner Bio-One, Kremsmünster, Austria) were pre-treated with celgosivir for 2 h and then infected with PRVABC59 at MOI 1 in the presence of celgosivir at concentrations ranging from 0.01 to 1000 μM or vehicle. Controls for media color, cells, and virus were included. At 48 and 72 hpi, Caspase-Glo^®^ Reagent was added, and after 30 min the luminescence of each sample was read five times using a Spark multimode microplate reader (TECAN, Männedorf, Switzerland) at an interval of 2 min for 15 s. The average of the five readings was used as the measurement for each biological replicate.

### Measurement of Necrosis

Cell necrosis was evaluated by measuring the levels of lactate dehydrogenase (LDH) in cells using the CytoTox 96 Non-Radioactive Cytotoxicity Assay (Promega) following the manufacturer’s instructions. LDH is a soluble stable cytosolic enzyme present in many cell types which is rapidly released into the cell culture medium following disruption of the plasma membrane. LDH is a widely used marker to measure cellular cytotoxicity and necrosis. CHME3 cells in 96-well cell culture plates were pre-treated with celgosivir for 2 h and then infected with PRVABC59 at MOI 1 in the presence of celgosivir at concentrations ranging from 0.01 to 1000 μM or vehicle. Controls for media color, cells, and virus were included. At 48 and 72 hpi, 50 μl of supernatant from each well was transferred into a new 96-well plate, and 50 μl of CytoTox 96^®^ Reagent was added to each well. After incubating at room temperature in the dark, 50 μl of Stop Solution was added to each well, and the optical density was measured at 490 nm using an ELx800 microplate reader (BioTek).

### Antiviral Gene Expression

CHME3 cells grown in 6-well plates and at 70% confluency were infected with PRVABC59 at MOI 1. After pre-incubation with the virus for 2 h, medium from cells was removed, cells were washed with PBS, and fresh infection medium containing 1 μM celgosivir was added. Negative control wells were added with infection medium without celgosivir. Three wells were used for each treatment. At 24, 48, and 72 h, total RNA was extracted from cells using the RNeasy Plus Minikit (Qiagen, Hilden, Germany) according to the manufacturer’s protocol. Complementary DNA was synthesized from 1 μg of total RNA using 4 μl of qScript cDNA SuperMix (5x) (QuantaBio, Beverly, MA, United States). qRT-PCR was performed on a 7500 Fast Real-Time PCR System (Applied Biosystems) using PowerSybr Green PCR MasterMix (Applied Biosystems) according to the manufacturer’s instructions. Expression of retinoic acid-inducible gene 1 (RIGI), melanoma differentiation-associated protein 5 (MDA5), interferon regulatory factor 7 (IRF7), interferon β (IFNβ), interferon-induced GTP-binding protein (MX1) and interferon-stimulated gene 15 (ISG15) were quantified, and hypoxanthine phosphoribosyltransferase 1 (HPRT1) was used as a house-keeping gene for data normalization. All primers used were previously published and were supplied by IDT (Megiorni et al., 2005; Kim et al., 2008; Nasirudeen et al., 2011; Muramatsu et al., 2014; Seng et al., 2014; Liu et al., 2015; Livingstone et al., 2015).

### Statistical Analysis

Prism 7 (GraphPad) was used to generate graphs and perform statistical analysis. Statistical comparison between data groups was performed using a two-tailed unpaired heteroscedastic *t*-test with Welch’s correction, with α = 0.05.

## Results

### No Cytotoxic Effects Were Observed in Vero or CHME3 Cells Treated With Iminosugars Up to a Concentration of 100 μM

**FIGURE 1:**
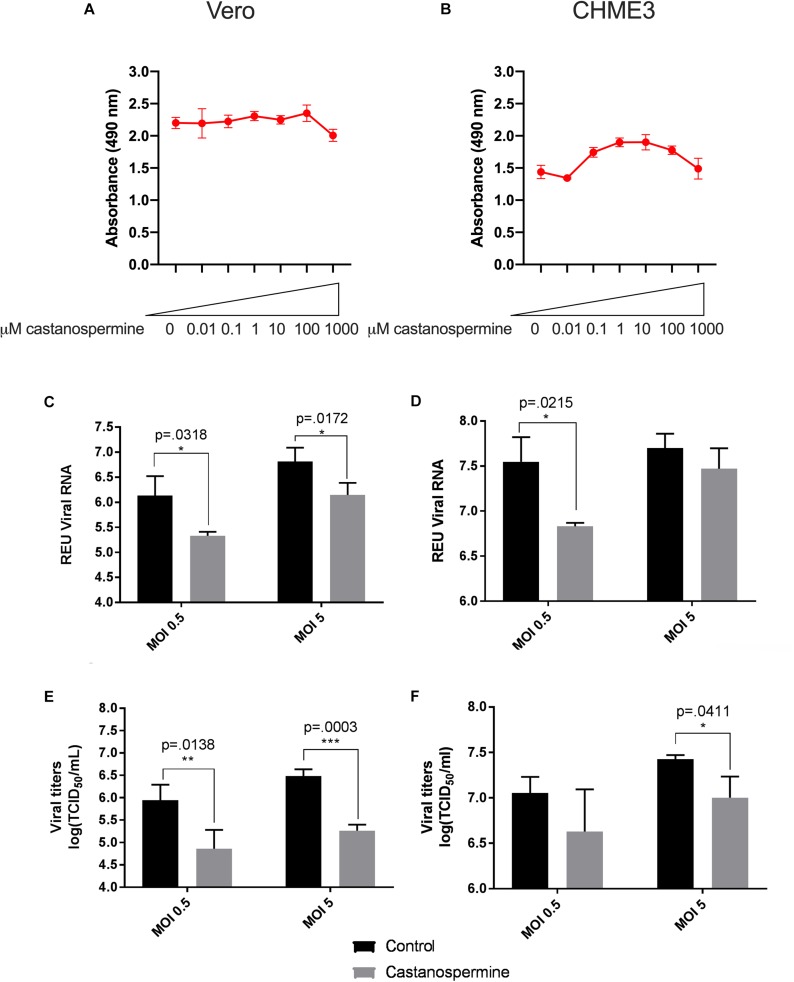
Castanospermine significantly inhibits Zika virus (ZIKV) replication in Vero and CHME3 cells without cytotoxicity. Vero cells **(A)** and CHME3 cells **(B)** were treated with castanospermine in concentrations ranging from 0.01 to 1000 μM, and cytotoxicity was quantified by MTS assay at 72 h post treatment. Absorbance was measured at 490 nm. Cell culture supernatant from Vero cells **(C,E)** and CHME3 cells **(D,F)** infected with ZIKV PRVABC59 at MOI of 0.5 or 5 with and without 1 μM castanospermine treatment were harvested at 48 hpi. ZIKV RNA levels were determined by qRT-PCR assay **(C,D)**. Infectious ZIKV titration was determined by TCID_50_ assay **(E,F)**. Data represent mean values of four biological replicates. Error bars represent standard deviation.

**FIGURE 2:**
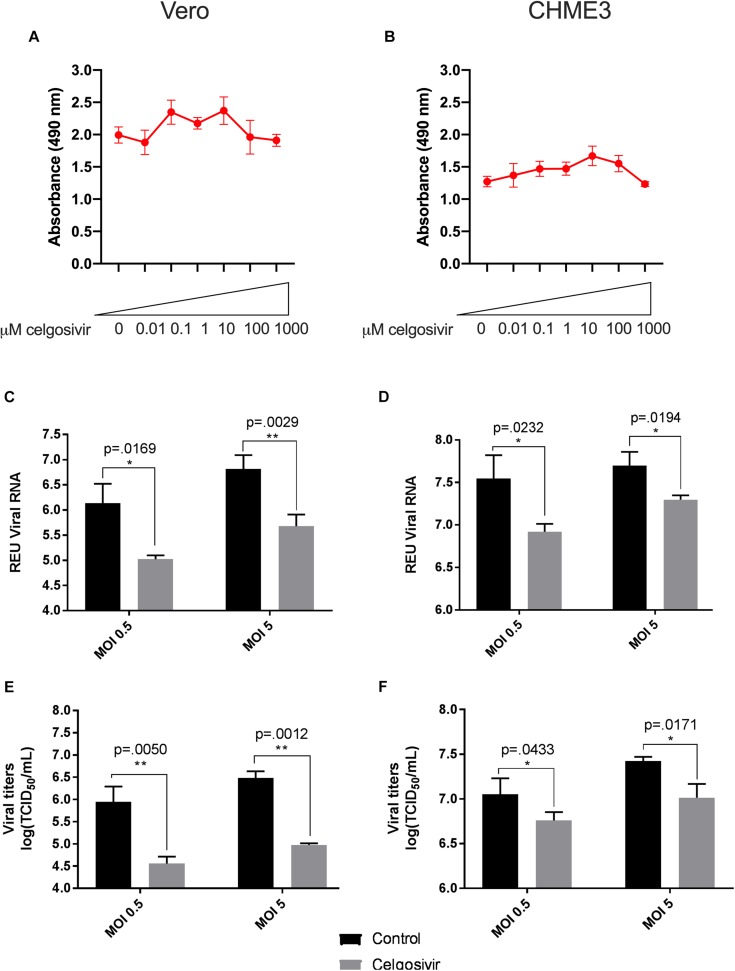
Celgosivir significantly inhibits Zika virus (ZIKV) replication in Vero and CHME3 cells without cytotoxicity. Vero cells **(A)** and CHME3 cells **(B)** were treated with celgosivir in concentrations ranging from 0.01 to 1000 μM, and cytotoxicity was quantified by MTS assay at 72 h post treatment. Absorbance was measured at 490 nm. Cell culture supernatant from Vero cells **(C,E)** and CHME3 cells **(D,F)** infected with ZIKV PRVABC59 at MOI of 0.5 or 5 with and without 1 μM celgosivir treatment were harvested at 48 hpi. ZIKV RNA levels were determined by qRT-PCR assay **(C,D)**. Data represent mean values of four biological replicates. Infectious ZIKV titration was determined by TCID_50_ assay **(E,F)**. Error bars represent standard deviation.

**FIGURE 3:**
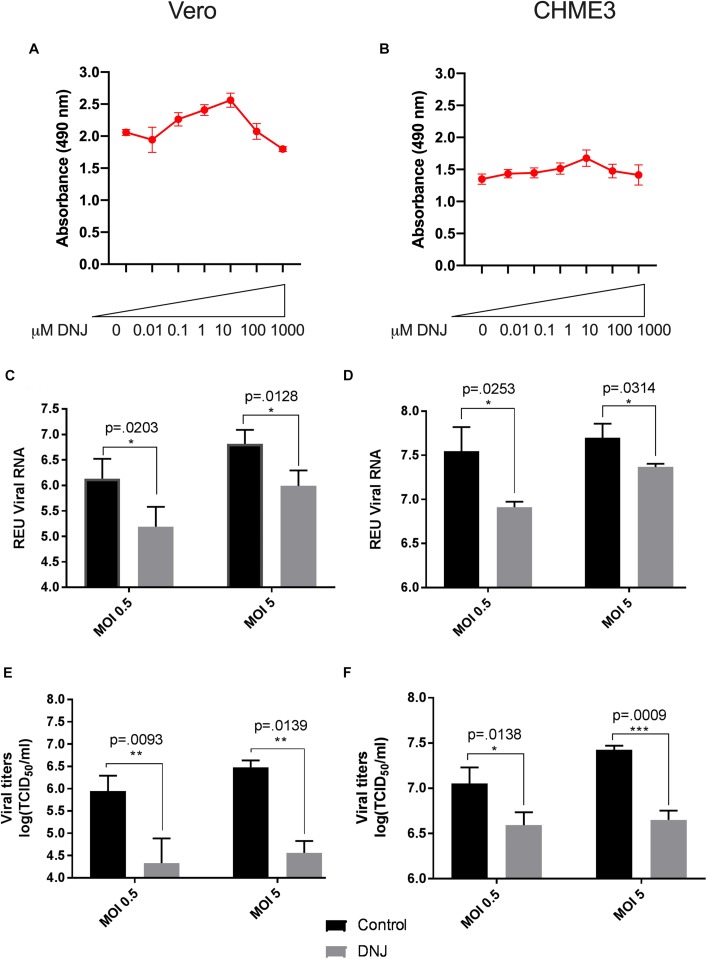
Deoxynojirimycin (DNJ) significantly inhibits Zika virus (ZIKV) replication in Vero and CHME3 cells without cytotoxicity. Vero cells **(A)** and CHME3 cells **(B)** were treated with DNJ in concentrations ranging from 0.01 to 1000 μM, and cytotoxicity was quantified by MTS assay at 72 h post treatment. Absorbance was measured at 490 nm. Cell culture supernatant from Vero cells **(C,E)** and CHME3 cells **(D,F)** infected with ZIKV PRVABC59 at MOI of 0.5 or 5 with and without 1 μM DNJ treatment were harvested at 48 hpi. ZIKV RNA levels were determined by qRT-PCR assay **(C,D)**. Infectious ZIKV titration was determined by TCID_50_ assay **(E,F)**. Data represent mean values of four biological replicates. Infectious ZIKV titration was determined by TCID_50_ assay **(E,F)**. Error bars represent standard deviation.

A total of three iminosugars, namely castanospermine, celgosivir, andDNJ, were tested for cytotoxicity in Vero and CHME3 cells at concentrations ranging from 0.01 to 1000 μM. The cytotoxicity screen was used to determine the range of concentrations which did not significantly reduce metabolically active cells and to define the appropriate conditions to use in further efficacy studies. No reduction in metabolic activity compared to vehicle control was found when castanospermine (Figures 1A,B), celgosivir (Figures 2A,B) or DNJ (Figures 3A,B) were applied to either cell type at concentrations ranging from 0.01 to 100 μM. However, a decrease in metabolic activity, indicating cytotoxicity, was observed in both cell types when treated with 1000 μM of castanospermine, celgosivir, or DNJ. The concentration of 1 μM was selected for targeted investigation of iminosugar effects on ZIKV infection.

### Iminosugar Treatment Significantly Inhibits ZIKV PRVABC59 Replication in Vero and CHME3 Cells

To evaluate the ability of iminosugars to inhibit ZIKV replication, we compared viral RNA and infectious virus in culture supernatants of ZIKV-infected Vero and CHME3 cells at 48 hpi with and without iminosugar treatment. Because the Asian lineage of ZIKV is responsible for the recent epidemics in the Americas with increased morbidity compared with ZIKV of the African lineage, we first investigated infection with the Asian-lineage strain PRVABC59, isolated in 2015 from a human patient in Puerto Rico. Significant reductions (*p* ≤ 0.0318) of PRVABC59 ZIKV RNA compared with vehicle control were observed in supernatants of Vero cells treated with 1 μM of castanospermine (Figure 1C), celgosivir (Figure 2C) or DNJ (Figure 3C). Similar reductions in viral RNA were seen between Vero cells infected at both MOI 0.5 and MOI 5. While a similar significant reduction (*p* ≤ 0.0253) of viral RNA was also observed in iminosugar-treated CHME3 cells infected at MOI 0.5 (Figures 1D, 2D, 3D), the reduction in viral RNA was not significant between castanospermine-treated and control cells infected at MOI 5 (Figure 1D).

Similarly, a statistically significant reduction (*p* ≤ 0.0433) in infectious PRVABC59 ZIKV titers as measured by TCID_50_ quantification was observed in castanospermine-, celgosivir- and DNJ-treated Vero (Figures 1E, 2E, 3E) and CHME3 cells (Figures 1F, 2F, 3F) infected with PRVABC59 ZIKV at both MOI 0.5 and MOI 5, with the exception of a reduction in castanospermine-treated CHME3 cells infected at MOI 0.5 which was not statistically significant.

### ZIKV PRVABC59 Infection Increases Antiviral Gene Expression in CHME3 Cells

**FIGURE 4:**
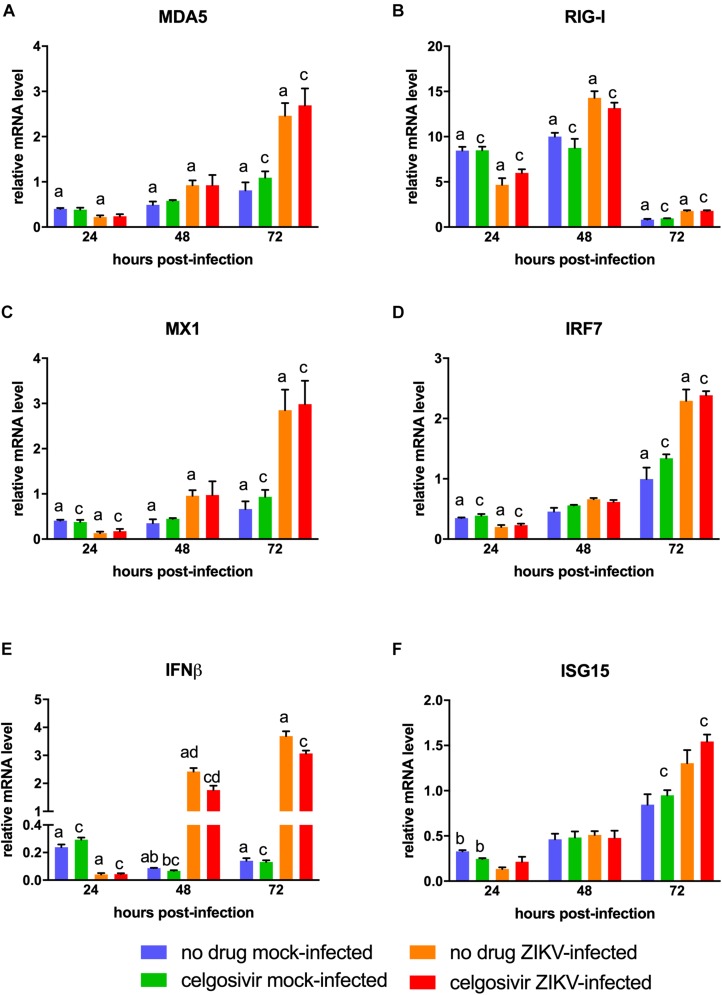
Celgosivir treatment does not inhibit antiviral response in CHME3 cells infected with Zika virus (ZIKV). Relative mRNA expression profiles of MDA5 **(A)**, RIG-I **(B)**, MX1 **(C)**, IRF7 **(D)**, IFNβ **(E)**, and ISG15 **(F)** of CHME3 cells infected with ZIKV PRVABC59 at 24, 48, and 72 hpi. Data points represent mRNA levels normalized to HPRT1 mRNA for each of three biological replicates. Error bars represent standard error of the mean. Data points with shared letters at the same timepoint have a statistically significant difference (*p* < 0.05) for the indicated gene.

The antiviral gene response to ZIKV in human microglial cells is not fully characterized. To understand the antiviral response in the CHME3 human microglial cell line, we next investigated the expression levels of antiviral genes MDA5, RIG-I, MX1, IRF7, IFNβ, and ISG15 following infection with PRVABC59 ZIKV (Figure 4). All genes were slightly downregulated at 24 hpi. MDA5, RIG-I, MX1, IFNβ were significantly upregulated in PRVABC59 ZIKV-infected cells compared with mock-infected cells at 48 and 72 hpi. IRF7 and ISG15 levels were also higher in infected cells at these timepoints, but the increase expression level was not statistically significant. The difference in antiviral gene expression levels between PRVABC59 ZIKV-infected and mock-infected cells was most evident with IFNβ (Figure 4E), MDA5 (Figure 4A) and MX1 (Figure 4C).

### Celgosivir Treatment Does Not Alter Antiviral Gene Response in CHME3 Cells

Iminosugars are known to reduce virus replication by inhibiting viral glycoprotein maturation and not by altering the cellular antiviral response. Therefore, antiviral gene expression analysis was also conducted on PRVABC59 ZIKV-infected CHME3 cells with or without treatment with 1 μM celgosivir. Celgosivir treatment alone did not significantly affect expression of investigated genes at most timepoints, and expression levels of MDA5, RIG-I, MX1, IRF7, and ISG15 were not significantly different between celgosivir-treated and vehicle-treated PRVABC59 ZIKV-infected cells at any timepoint (Figure 4). At 48 hpi, a slight decrease (22%) of IFNβ gene expression was observed between celgosivir-treated and untreated mock-infected CHME3 cells, which was statistically significant. However, the level of IFNβ expression was again similar at 72 hpi. Similarly, IFNβ expression levels were decreased by 27% (*p* = 0.033) in PRVABC59 ZIKV-infected celgosivir-treated cells compared with ZIKV-infected mock-infected cells at 48 hpi. For all genes in cells treated with celgosivir, the expression levels in virus-infected cells were significantly higher than in mock-infected cells at 72 hpi.

### Celgosivir Treatment Protects Cells From ZIKV Cytopathogenicity

**FIGURE 5:**
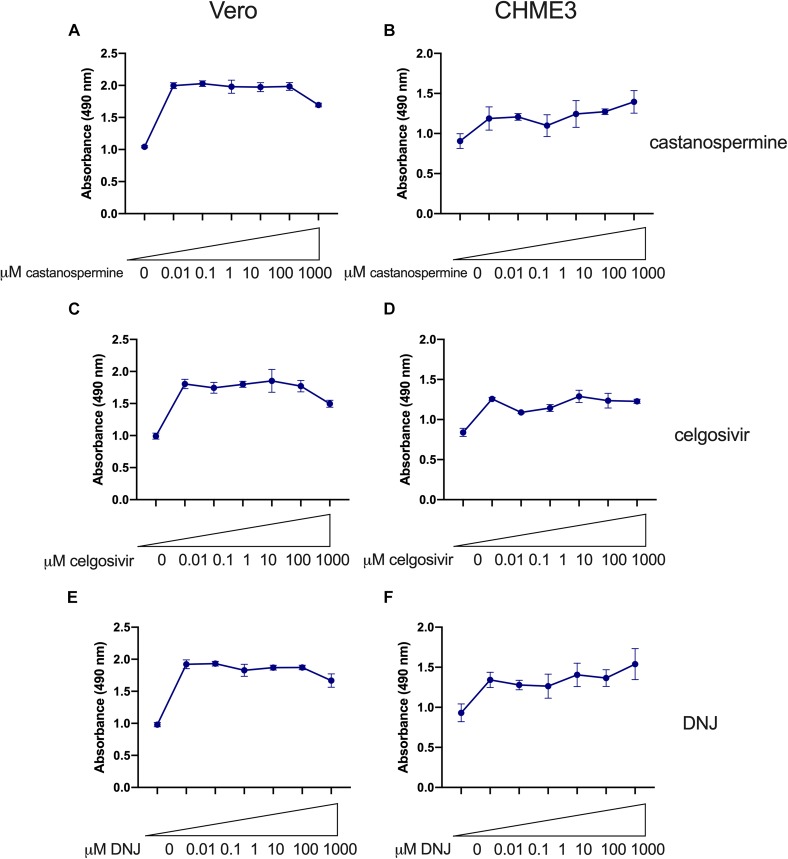
Iminosugar endoplasmic-reticulum α-glucosidase inhibitor treatment restores cell viability following infection with Zika virus (ZIKV) in Vero and CHME3 cells. Cell viability at 72 h post infection as measured by MTS assay of castanospermine- **(A,B)**, celgosivir- **(C,D)**, and deoxynojirimycin (DNJ)- **(E,F)** treated or vehicle-treated Vero **(A,C,E)** or CHME3 **(B,D,F)** cells infected with ZIKV strain PRVABC59. Absorbance was measured at 490 nm. Data represents mean absorbance of four biological replicates, with error bars showing standard deviation.

Having demonstrated that the inhibition of PRVABC59 ZIKV by iminosugar celgosivir was not due to altered cellular antiviral gene expression, all three iminosugars were further evaluated for antiviral activity. Castanospermine (Figures 5A,B), celgosivir (Figures 5C,D) and DNJ (Figures 5E,F), were similar in their ability to reduce ZIKV-induced cell death induced by PRVABC59 ZIKV in both Vero and CHME3 cells. Across all concentrations, 1 or 10 μM of each compound was the most effective in reducing ZIKV-induced cell death. Vero cells infected with PRVABC59 alone resulted in 50–55% cell death 72 h post-infection as reflected by a decrease in absorbance. Uninfected cells showed an absorbance close to 2.0 Au at 490 nm, whereas the absorbance of PRVABC59 infected cells was 1.0 Au. However, PRVABC59-infected cells treated with iminosugars ranging from 0.01 to 1000 μM were able to rescue the extent of cell death by 30–40% (Figures 5A,C,E). In CHME3 cells infected with PRVABC59 ZIKV, similar trends were seen. Generally, PRVABC59 ZIKV-infected CHME3 cells alone underwent 21–45% cell death. Iminosugars were able to rescue the number of viable cells by ∼40% in comparison to PRVABC59 ZIKV-infected CHME3 cells alone (Figures 5B,D,F). Overall, the ability to rescue cell death was comparable between all iminosugars tested.

### Celgosivir Treatment Does Not Affect Apoptosis in ZIKV Infected CHME3 Cells

**FIGURE 6:**
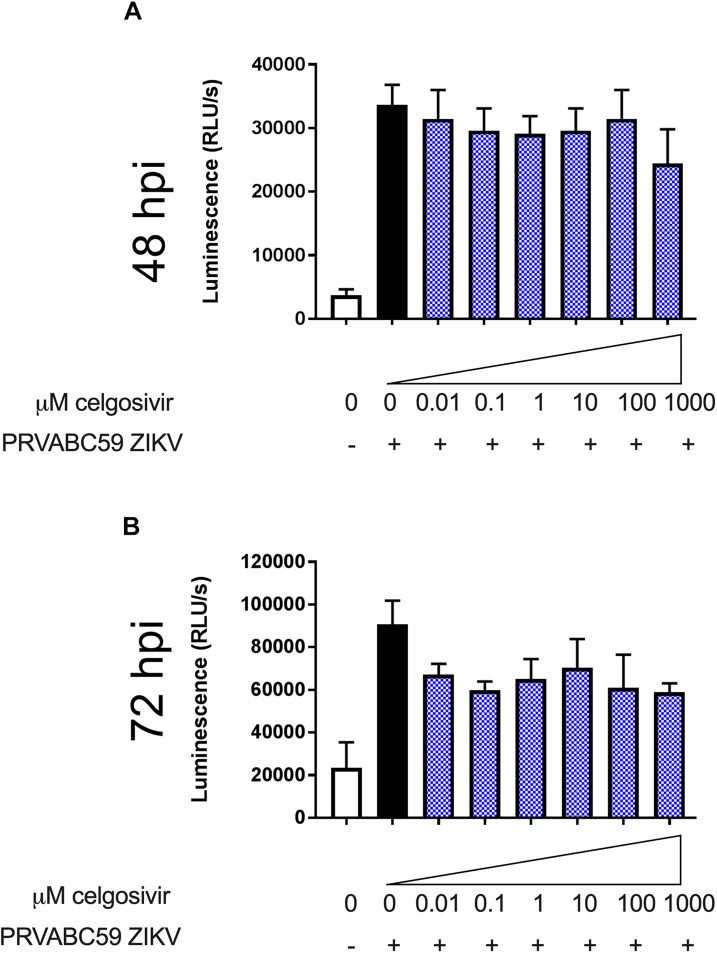
Celgosivir does not alter activated caspase 3/7 levels in CHME3 cells infected with Zika virus (ZIKV). Luminescence measurements of activated caspase 3/7 from CHME3 cells 48 **(A)** and 72 **(B)** hours following infection with ZIKV PRVABC59 with and without celgosivir. Data points represents mean luminescence of four biological replicates with error bars showing standard deviation.

Zika virus has been shown to induce apoptosis in different sets of cells, such as fetal neural progenitor cells, cranial neural crest cells, peripheral neurons, dendritic cells and microglia (Manangeeswaran et al., 2016; Souza et al., 2016; Russo and Beltrao-Braga, 2017; Tiwari et al., 2017; Swartwout et al., 2017; Wen et al., 2017; Yuan et al., 2017). Apoptosis is a mechanism the cells use to limit ZIKV spread. Levels of activated caspases 3 and 7 were measured to investigate the if iminosugar treatment could affect the apoptosis response in PRVABC59 ZIKV-infected cells. CHME3 infection with ZIKV strain PRVABC59 at MOI 1 substantially increased activated caspases 3 and 7 levels at 48 and 72 hpi (Figure 6). At concentrations ranging from 0.01 to 1000 μM, celgosivir did not significantly reduce levels of activated caspases 3 and 7 in comparison to untreated CHME3 cells infected with PRVABC59 ZIKV at both 48 and 72 hpi (Figure 6).

### Celgosivir Treatment Is Associated With Lower Cell Necrosis After ZIKV Infection

**FIGURE 7:**
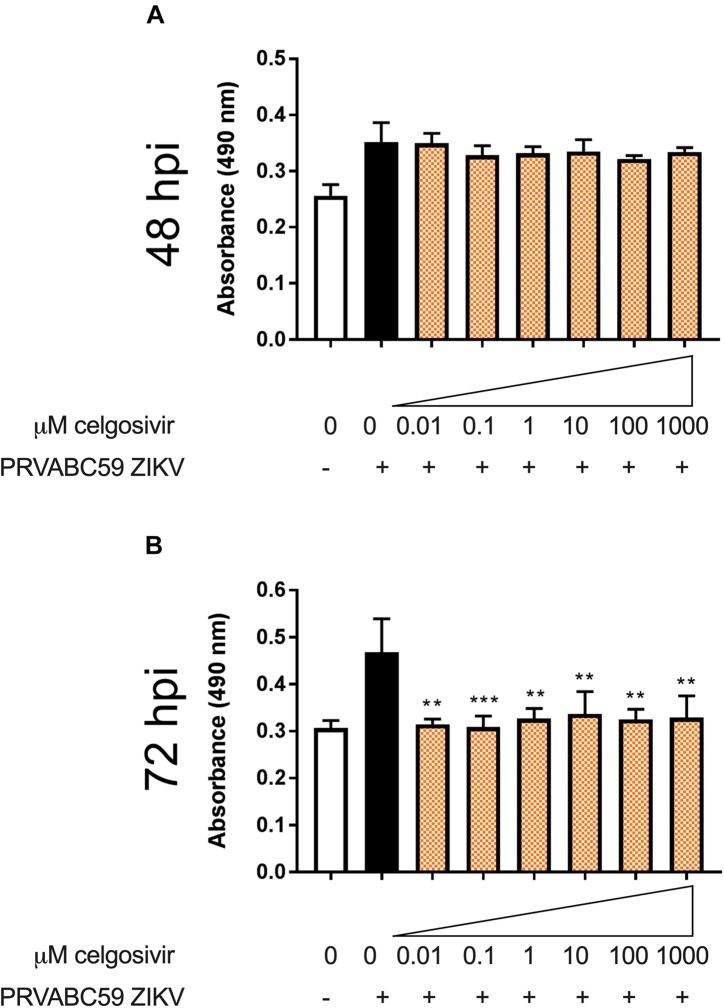
Celgosivir rescues CHME3 cells from necrosis following infection with Zika virus (ZIKV). Absorbance measurements of LDH levels from CHME3 cells 48 **(A)** and 72 **(B)** hours following infection with ZIKV PRVABC59 at MOI of 1 with and without celgosivir. Data points represents mean absorbance of four biological replicates with error bars showing standard deviation. **p < 0.01, ***p < 0.001.

Zika virus has been shown to cause necrosis in human brain microvascular endothelial cells, especially at 48, 72, and 96 hpi (Papa et al., 2017). Histopathological changes such as necrosis have been observed in the brains of newborn babies who are congenitally infected with ZIKV (Roosecelis Brasil Martines et al., 2016). The effect of iminosugars on cytopathogenicity was further characterized by a lactate dehydrogenase (LDH) activity assay to determine if iminosugar use could reduce ZIKV-induced necrosis. CHME3 infection with PRVABC59 ZIKV alone substantially increased LDH levels at 48 and 72 hpi (Figure 7). Treatment of ZIKV-infected cells with 1 μM celgosivir did not reduce levels of LDH at any observed concentration at 48 hpi (Figure 7A). However, at 72 hpi celgosivir reduced PRVABC59 ZIKV-induced LDH activity to levels equivalent to mock-infected cells (Figure 7B).

### Iminosugars Inhibit Replication of Multiple ZIKV Strains

**FIGURE 8:**
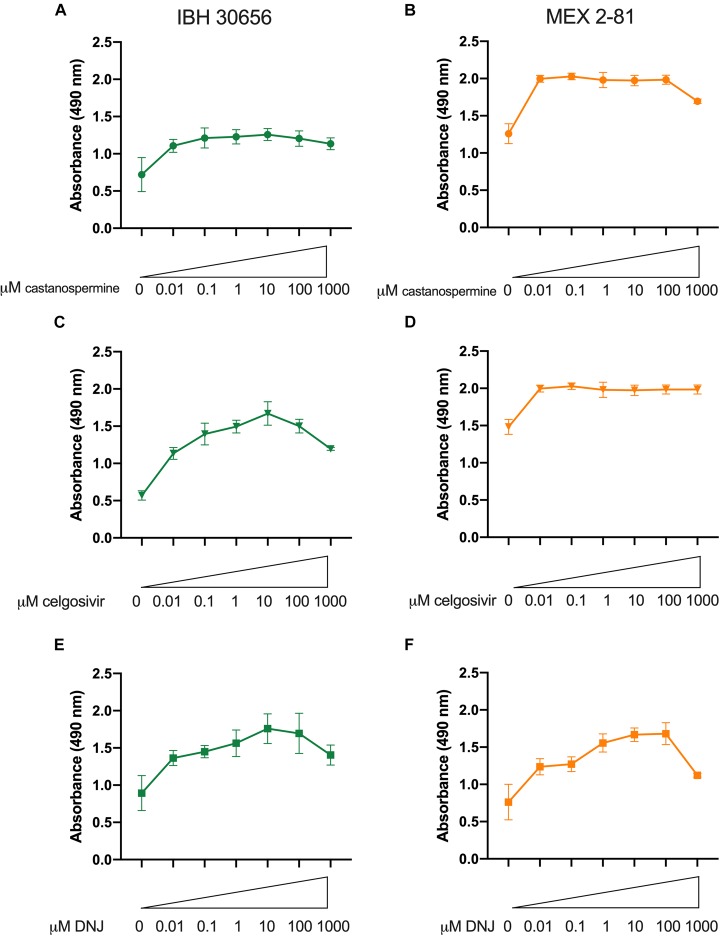
Iminosugar endoplasmic-reticulum α-glucosidase inhibitor treatment restores cell viability following infection Zika virus (ZIKV) strains IBH30656 or MEX 2-81 in Vero cells. Cell viability at 72 h post infection as measured by MTS assay of castanospermine- **(A,B)**, celgosivir- **(C,D)**, and DNJ- **(E,F)** treated or vehicle-treated Vero cells infected with ZIKV strain IBH30656 **(A,C,E)** or MEX 2-81 **(B,D,F)**. Absorbance was measured at 490 nm. Data represents mean absorbance of four biological replicates, with error bars showing standard deviation.

Iminosugars were further evaluated for antiviral activity against two other ZIKV isolates in Vero cells by measuring reduction of cytopathic effect (cell death) after ZIKV infection. An African lineage strain isolated from a human patient in Nigeria in 1968, IBH30656, and an Asian lineage strain isolated from an *Aedes aegypti* mosquito in Mexico in 2016, MEX 2-81, were used to infect cells Vero cells. When cells were treated with iminosugar, ZIKV-induced cell death was reduced regardless of the ZIKV strain or cell type used (Figure 8). Vero cells infected with IBH30656 or MEX 2-81 showed 50–55% reduction in absorbance and therefore cell death at 72 hpi, and iminosugars ranging from 0.01 to 1000 μM increased absorbance and therefore reduced the extent of cell death by 30–40% (Figure 8).

## Discussion

Zika virus continues to spread throughout the world and remains a significant public health threat. While multiple intervention therapies are under development, currently there are no approved vaccines or antivirals to prevent damaging neurological syndromes such as Guillain-Barré syndrome and microcephaly, which are multifactorial diseases that still need to be studied in depth. Hence, there is an urgent need to investigate and evaluate antiviral therapies for ZIKV infection. In this study, we evaluated the ability of three iminosugars with ER α-glucosidase inhibitor (ER-AGI) activity with different chemical structures to inhibit ZIKV replication both in a cell line commonly used to propagate ZIKV (Vero cells) and in a clinically relevant human microglial cell line (CHME3 cells). This study provides evidence of effectiveness of iminosugars to inhibit ZIKV in cells from the brain, rescuing cells from ZIKV-induced cell death while maintaining cellular antiviral response.

We found that following ZIKV infection in both Vero and CHME3 cells infected at MOI 1, as little as 0.01 μM of either celgosivir or DNJ improved cell viability to over 90%. Castanospermine treatment was slightly less effective, requiring 10 μM to achieve 90% cell viability in CHME3 cells. A recent study reported the first use of iminosugar therapy against ZIKV wherein the iminosugar IHVR-19029 decreased ZIKV RNA production following HEK293 embryonic kidney cells infected at MOI 0.1 when used at 0.1 μM, the lowest concentration tested (Ma et al., 2018). Earlier studies have shown that iminosugars are able to inhibit multiple flaviviruses though with differing effectiveness. For example, Rathore et al. showed that celgosivir and castanospermine could inhibit dengue virus (DENV) in BHK-21 cells infected at MOI 0.3, but celgosivir was 100 times more effective than castanospermine (Rathore et al., 2011). The half maximal effective concentration of celgosivir was similar against all DENV serotypes (DENV 1-4) in THP-1 human monocytes (Rathore et al., 2011), and castanospermine has previously been shown to inhibit viral spread and virion secretion of all four serotypes of DENV (Whitby et al., 2005).

Although the iminosugars we tested demonstrated effectiveness against ZIKV starting at very low concentrations, castanospermine, DNJ and celgosivir were well-tolerated in Vero and CHME3 cells even at concentrations of 100 μM or more. This low cytotoxicity profile of iminosugars has also been observed in many other cell types including HEK293 cells with IHVR-19029 (Ma et al., 2018), monocyte-derived macrophages with DNJ (Sayce et al., 2016) and bovine MDBK cells with castanospermine, celgosivir and DNJ, among others (Whitby et al., 2004). Lack of cytotoxicity specifically in CHME3 microglial cells is an attractive feature of these iminosugars for potential use against ZIKV to prevent damage to the developing brain.

Iminosugars are known to interfere with viral protein synthesis as well as the maturation of viral particles (Chang et al., 2013a). The results of the current study are consistent with other published data showing the potential of iminosugars as potent antivirals not only against flaviviruses but other enveloped viruses (Schlesinger et al., 1985; Datema et al., 1987; Taylor et al., 1988; Fischl et al., 1994; Wu et al., 2002; Simsek et al., 2006; Gu et al., 2007; Qu et al., 2011; Chang et al., 2013a, b; Perry et al., 2013; Ma et al., 2018). Flaviviruses are incredibly reliant on ER alpha-glucosidases I and II for N-linked oligosaccharide trimming of their glycoproteins, which allows for subsequent interactions with ER chaperones calnexin and calreticulin (Ma et al., 2018). ER α-glucosidase I removes the terminal α1,2-linked glucose from Glc3Man9GlcNAc2, and α-glucosidase II removes the second and third glucose before further processing to become a mature virion (Kim et al., 2017). We found that iminosugar treatment resulted both in reduced viral RNA and lower infectious virus in cell culture supernatant compared with vehicle-treated cells, indicating impaired virus replication leading to production of fewer virus particles. Notably, the reduction in infectious virus titer was much higher than the reduction in viral RNA. This observation suggests that there are also defective particles suggesting inefficient virus maturation in iminosugar-treated cells. Viral RNA measured by qRT-PCR that is not accounted for by infectious virus particles likely represents viral RNA encapsulated in non-infectious virions (Sayce et al., 2016). While our experiments did not investigate the mechanism underlying the ability of iminosugars to inhibit ZIKV replication, our results suggest that the reduction in ZIKV-induced cell death by iminosugars occurs through reducing the secretion of infectious virus particles or by secreting particles that are less infectious.

It is well-known that the infection of cells with ZIKV induces the upregulation of antiviral genes (Carlin et al., 2018). Our study also found that in CHME3 cells ZIKV infection resulted in upregulation of type I interferon (IFNβ), interferon-stimulated genes (MX-1 and ISG15), pattern recognition receptors (RIG-I and MDA5) and IRF7. This upregulation was not significantly affected by addition of celgosivir, despite celgosivir reducing magnitude of ZIKV infection. Since the results of misfolded viral proteins includes not only reduced successful virus assembly and maturation but also protein accumulation in the ER and eventual ERAD, iminosugar treatment can induce ER stress and the unfolded protein response (Zakaria et al., 2018). Such a response can lead to stimulation of antiviral innate immune response pathways as well as apoptosis to limit viral infection (Smith, 2014). We found a significant increase in activated caspase 3/7 in ZIKV-infected CHME3 cells compared with mock-infected cells at 48 and 72 hpi, indicating significant activation of apoptosis in virus-infected cells. Our findings are in accordance with an earlier report that showed significant activation of caspases in neural progenitor cells at 24 h after infection with ZIKV (Souza et al., 2016). Notably, there was no statistically significant difference in the levels of caspase 3/7 between celgosivir- and vehicle-treated cells after ZIKV infection. The significance of apoptosis of microglia is unclear, particularly in the developing brain. Primary astrocytes, the cell type that is most abundant in developing brain (Reemst et al., 2016), that supported persistent ZIKV infection of both African and Asian lineage ZIKV were relatively resistant to virus-induced apoptosis, suggesting that lack of apoptosis could lead to persistence of ZIKV infection in developing brain cells and lead to ZIKV-induced damage such as microcephaly (Limonta et al., 2018).

A key pathogenicity factor in viral infection is necrosis of virus-infected cells, which leads to pro-inflammatory response due to the leakage of cellular contents. It is clear that ZIKV can induce high levels of cell death (Costa et al., 2017; Olmo et al., 2017) and that cell death ultimately drives ZIKV-related developmental abnormalities of the brain. As macrophages, microglia are the main immune cell of the brain and begin this role early in brain development (Gomez Perdiguero et al., 2015). Our data showed that ZIKV infection of CHME3 microglial cells results in substantial necrosis as evidenced by significantly elevated LDH levels. Further, we found that celgosivir was able to decrease necrosis induced by ZIKV at 72 hpi, which could limit inflammatory responses *in vivo* and help limit the clinical disease associated with ZIKV infection. That treatment of ZIKV-infected CHME3 microglial cells with iminosugars reversed the decrease in metabolic activity and reduced percentage of viability due to ZIKV infection. If similar responses occurred with microglial cells *in vivo*, more cells in the developing brain may remain alive, the remaining microglia could help sustain an effective response fostering eradication of ZIKV from brain. Finally, since there is only one subtype of ZIKV, we hypothesized that iminosugars would cause similar antiviral effects against different ZIKV isolates. The results of this study were in strong support of our hypothesis as a decrease in cytopathogenicity was seen upon the addition of castanospermine, celgosivir, and DNJ, regardless of the ZIKV strain used. These isolates represent strains isolated from 1968 to 2016 from different geographic locations, time periods, hosts and lineages, and results show that iminosugars similarly inhibit cell death induced by different isolates.

Of all compounds studied, celgosivir is the most promising as evidenced by its ability to reduce viral RNA and infectious virus titers in both Vero and CHME3 cells with no effect on cellular apoptosis and antiviral responses. Celgosivir (or 6-*O*-butanoyl castanospermine) is alkylated on its ring nitrogen and has been shown to be more effective as a chemical target than its parent compound or compounds that have no modification on their ring nitrogen (Sayce et al., 2016). Celgosivir has been considered in human clinical trials against hepatitis C virus, HIV and dengue virus (Durantel, 2009; Sung et al., 2016). The dosing and delivery schedule seems to be a critical aspect of successful use of celgosivir as an antiviral treatment in humans (Sung et al., 2016; Watanabe et al., 2016). Its highly protective ability against dengue virus in very vulnerable mice with disrupted type I and type II interferon response is reason to believe that an optimized dosing regimen will allow celgosivir to succeed in clinical trials against dengue virus (Rathore et al., 2011; Watanabe et al., 2012). The results of our study show that celgosivir may be a promising iminosugar to treat ZIKV and potentially prevent microcephaly. In light of celgosivir’s action against ZIKV, future studies may also evaluate the dose- and schedule-dependent effectiveness of celgosivir against this related flavivirus.

We have shown here three iminosugars that are active against ZIKV in a dose-dependent manner *in vitro*. These results are encouraging and are essential as a starting point for further validation of *in vivo* mouse models. In conclusion, the results of this study increase the possibility that iminosugars may be a promising therapeutic against ZIKV infections.

## Data Availability Statement

The datasets generated for this study are available on request to the corresponding author.

## Author Contributions

SK conceived of the study and designed the experiments. GB and LL conducted the experiments. GB, RN, and SK analyzed the data and wrote the manuscript. IB and SC contributed to the experiments and data analysis.

## Conflict of Interest

The authors declare that the research was conducted in the absence of any commercial or financial relationships that could be construed as a potential conflict of interest.
