# Supplementary material for: Iminosugars With Endoplasmic Reticulum α-Glucosidase Inhibitor Activity Inhibit ZIKV Replication and Reverse Cytopathogenicity in vitro
Source: Front Microbiol. 2020 Apr 7;11:531. doi: 10.3389/fmicb.2020.00531 (PMC7179685; doi:10.3389/fmicb.2020.00531)
Supplement: Supplementary file 1 [file Data_Sheet_1.docx]

Supplementary Material

## Supplementary Figures

**
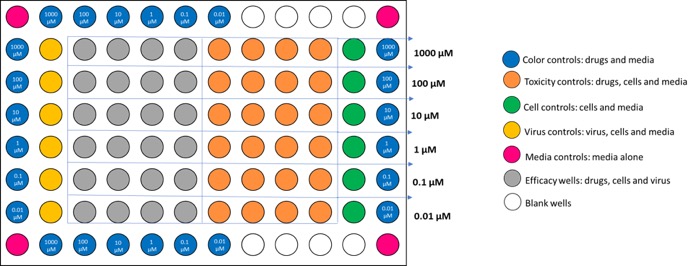
**

**Supplementary Figure 1.** **Plate layout for testing cytotoxicity and efficacy of iminosugar endoplasmic-reticulum α-glucosidase inhibitors (ER-AGIs) against Zika virus.**

.
